# Tissue-specific conditional PKCε knockout mice: a model to precisely reveal PKCε functional role in initiation, promotion and progression of cancer

**DOI:** 10.18632/oncotarget.8850

**Published:** 2016-04-20

**Authors:** Bilal Bin Hafeez, Louise Meske, Ashok Singh, Anupama Singh, Weixiong Zhong, Patricia Powers, Manorama John, Anne E. Griep, Ajit K. Verma

**Affiliations:** ^1^ Department of Human Oncology, Wisconsin Institute for Medical Research, Paul Carbone Comprehensive Cancer Center, School of Medicine and Public Health, University of Wisconsin, Madison, WI 53705, USA; ^2^ Department of Pathology, Wisconsin Institute for Medical Research, Paul Carbone Comprehensive Cancer Center, School of Medicine and Public Health, University of Wisconsin, Madison, WI 53705, USA; ^3^ University of Wisconsin Biotechnology Center, Wisconsin Institute for Medical Research, Paul Carbone Comprehensive Cancer Center, School of Medicine and Public Health, University of Wisconsin, Madison, WI 53705, USA; ^4^ Department of Cell and Regenerative Biology, Wisconsin Institute for Medical Research, Paul Carbone Comprehensive Cancer Center, School of Medicine and Public Health, University of Wisconsin, Madison, WI 53705, USA

**Keywords:** PKCε^LoxP/LoxP^ mice, transgenic mice

## Abstract

PKCε is a transforming oncogene and a predictive biomarker of various human cancers. However, a precise in vivo link of PKCε to cancer induction, progression and metastasis remain undefined. To achieve these goals, we generated tissue specific conditional PKCε knockout mice (PKCε-CKO) using cre-lox technology. Homozygous PKCε^LoxP/LoxP^ mice have normal body weight and phenotype. To determine what effect loss of PKCε would have on the prostate, the PKCε^LoxP/LoxP^ mice were bred to probasin cre (PB-Cre4^+^) mice which express cre specifically in the prostate epithelium of postnatal mice. Western blot and immunohistochemical analyses showed reduced levels of PKCε specifically in the prostate of PKCε-CKO mice. Histopathological analyses of prostate from both PKCε^LoxP/LoxP^ and prostate PKCε-CKO mice showed normal pathology. To determine the functional impact of prostate specific deletion of PKCε on prostate tumor growth, we performed an orthotopic xenograft study. Transgenic adenocarcinoma of the mouse prostate (TRAMP) cells (TRAMPC1, 2×10^6^) were implanted in the prostate of PKCε-CKO mice. Mice were sacrificed at 6th week post-implantation. Results demonstrated a significant (P<0.05) decrease in the growth of TRAMPC1 cells-derived xenograft tumors in PKCε-CKO mice compared to wild type. To determine a link of PKCε to ultraviolet radiation (UVR) exposure-induced epidermal Stat3 phosphorylation, PKCε^LoxP/LoxP^ mice were bred to tamoxifen-inducible K14 Cre mice. PKCε deletion in the epidermis resulted in inhibition of UVR-induced Stat3 phosphorylation. In summary, our novel PKCε^LoxP/LoxP^ mice will be useful for defining the link of PKCε to various cancers in specific organ, tissue, or cells.

## INTRODUCTION

PKC is a major intracellular receptor for the mouse skin tumor promoter 12-O-tetradecanoylphorbol-13-acetate. PKC represents a large family of phosphatidylserine (PS)-dependent serine/threonine kinases [1–5]. PKCε is among the novel PKC isoforms (δ, ε, η and θ) which retain responsiveness to PS, but do not require Ca^2+^ for full activation [1–3]. PKCε is involved in regulation of diverse cellular functions such as neoplastic transformation, cell adhesion, mitogenicity and cell invasion [6, 7].

Overwhelming evidence from our laboratory and others indicates that PKCε is a transforming oncogene and a predictive biomarker of various human cancers including prostate, breast, head and neck, lung, brain, bladder and cutaneous squamous cell carcinoma [8–15]. Specific examples indicating the role of PKCε in the development of prostate and cSCC are cited. For example, overexpression of PKCε is sufficient to promote conversion of androgen-dependent (AD) LNCaP cells to androgen-independent (AI) variant, which rapidly initiates tumor growth in vivo in both intact and castrated athymic nude mice [16]. Overexpression of PKCε protected LNCaP cells against apoptotic stimuli via inducing phosphorylation of Bad at Ser112 [17]. It has been shown that integrin signaling links PKCε to the PKB/Akt survival pathway in recurrent prostate cancer (PCa) cells [18]. Proteomic analysis of PCa CWR22 cells xenografts show that association of PKCε with Bax may neutralize apoptotic signals propagated through the mitochondrial death-signaling pathway [19]. We and others have previously shown that PKCε level correlates with the aggressiveness of human PCa. Also, PKCε is overexpressed in PCa spontaneously developed in transgenic adenocarcinoma of the mouse prostate (TRAMP) mice, an autochthonous transgenic model that perfectly mimics to the human disease [12]. We have also shown that PKCε is a protein partner of transcription factor Stat3. PKCε associates with Stat3 and this association increases with the progression of the diseases in TRAMP mice and in human PCa [12]. Taken together, all of these findings suggest that PKCε is an oncogene and is involved in PCa development, aggressiveness, as well as in the emergence of AI PCa.

An experimental approach to define mechanism by which PKCε signals biological effects involves inactivation of PKCε. Several approaches that are employed to inactivate PKCε include germline PKCε knockout mice, overexpression of kinase-inactive mutant, cell permeable peptide, pharmacological inhibitors and siRNA [20]. A major limitation in these strategies is cell specificity [20]. We have shown that genetic loss of PKCε in TRAMP mice inhibits development and metastasis of PCa [12]. However, in this experiment germline PKCε knockout mice were used. These germline PKCε knockout mice are viable and lack phenotype. It is possible that the absence of a phenotype is due to compensatory mechanisms [20, 21]. To precisely determine the in vivo link of PKCε in a tissue specific manner at a given time point to cancer induction, progression and metastasis, we generated tissue-specific conditional PKCε knockout mice (PKCε-CKO).

We generated floxed PKCε mice using cre recombinase technology and crossed these mice to prostate specific cre (PB^Cre4/+^) and skin specific cre (K14^Cre/+^) mice to delete PKCε specifically in the prostate epithelium and epidermis respectively. Specific deletion of PKCε inhibited: 1) the growth of orthotopic allograft tumors developed by TRAMPC1 cell implantation in the prostate, and, 2) ultraviolet radiation exposure (2 kJ/m^2^)-induced Stat3 phosphorylation in the skin.

## RESULTS

### Generation of floxed PKCε targeting vector

**Figure 1:**
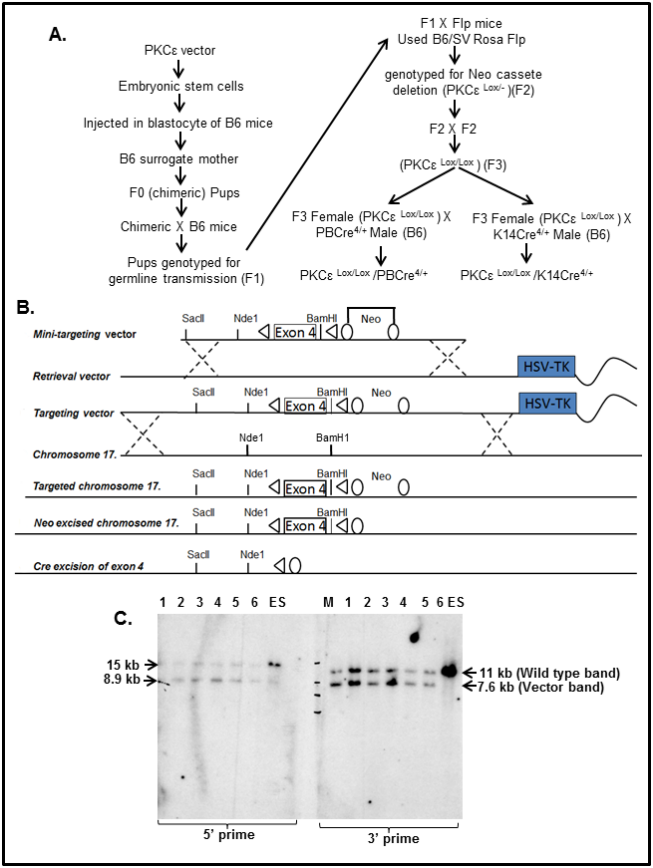
Generation of floxed PKCε (PKCε^LoxP/LoxP^) mice **A.** Schematic diagram showing generation and characterization of tissue specific PKCε knockout mice. **B.** Targeting vector diagram showing LoxP sites flanking Exon 4 of PKCε gene that are the targets of Cre recombinase enzyme and neo flanking with FRT. Exon 4 was deleted leaving a mutant form of PKCε allele and neo cassette was removed by FLP recombinase. **C.** Southern blot analysis to confirm incorporation of targeting vector containing LoxP-PKCε Exon 4-LoxP into the endogenous PKCε locus in embryonic stem (ES) cells. Briefly, DNA was taken from 6 representative wells (numbered 1-6 in the gel) of ES cells containing LoxP-PKCε Exon 4- LoxP targeting vector. On the left side of the blot, DNA was digested with Nde1 and probed with the 5′ flanking probe. The blot shows the wild type target allele at 15 kb and the targeted allele at 8.9 kb. On the right side of the blot, DNA was digested with BamH1 and probed with the 3′ flanking probe. The blot shows the wild type allele at 11 kb and the targeted allele at 7.6 kb. Abbreviations: M=molecular weight marker.

A schematic diagram for generation and characterization of floxed PKCε mice is illustrated in Figure 1A. The recombineering strategy which is a highly efficient phage-based Escherichia coli homologous recombination system was used to generate the PKCε targeting vector (Figure 1B). Specifically, loxP sites were introduced flanking exon 4 and an FRT flanked Neo cassette, for neomycin selection of transformed ES cells, was introduced 3′ to the LoxP site in intron 4. The 160 bp exon 4 was selected because removal of exon 4 will result in a frame shift and the premature truncation of the PKCε protein. The mini-targeting vector was cloned into HSV-TK retrieval vector to generate the PKCε targeting vector.

### PKCε vector targeting in ES cells

The floxed PKCε-targeting vector was electroporated into JM8A3 ES cells. These ES cells, being derived from C57BL/6N mice, have advantage to easily create transgenic mice directly onto a B6 background. Following electroporation, ES cells were grown in medium containing G418, to select ES cells in which the targeting vector had integrated and in gancyclovir (GANC) to select against cells in which the targeting vector had integrated into non-homologous sites. Neo and GANC resistant colonies were picked into a 96 well plate, and then triplated to give a master plate and two DNA plates for Southern blot analysis (Figure 1C). In brief, DNA was isolated from the ES cells colonies and digested with restriction enzymes BamH1 for hybridization to the 3′ probe and Nde1 for hybridization to the 5′ probe. These samples were electrophoresed on agarose gels, transferred to nylon membranes and hybridized with 5′ and 3′ labeled probes. Clones correctly targeted at the 5′ end were identified by the presence of 8.9 kb (targeted allele and 15kb (wild type allele) bands. Clones correctly targeted at the 3′ end were identified by the presence of 7.6 kb (targeted allele) and 11kb (wild type allele) bands. Six correctly targeted PKCε clones were selected for expansion and chromosome counting (Figure 1C).

### Chimeric founders (FO)

Two karyotypically normal euploid clones were micro-injected into C57BL/6 host blastocysts to produce chimeric founders. Pups carrying the targeted allele were identified by genotyping using primers sequences shown in Table 1.

**Table 1:** List of primer pairs used in the study for genotyping

PCR genotyping plan forfloxed PKCε mice	Primers sequence (5′-------3′)	PCR product
**Step 1.** PCR to identify pups carrying targeted allele (F0)	**Fwd:** GTA AGT CCC TGG AGA AGG GAG GGG GTT **Rev:** GGG CTC TAT GGC TTC TGA GGC	221bp (Targeted allele)
**Step 2.** F0 Backcrossed to C57Bl/6J to confirm gene transmission (F1)	**Fwd:** GTA AGT CCC TGG AGA AGG GAG GGG GTT **Rev:** GGG CTC TAT GGC TTC TGA GGC	221bp (Targeted allele)
**Step 3.** PCR to identify pups after Neo Cassette removal (F2). Neo is flanked by FRT	**Fwd:** GTA AGT CCC TGG AGA AGG GAG GGG GTT **Rev:** GAA CTC AGA GAC CCA CCC TCC	745bp (Targeted allele) 585 (Wild type)
**Step 4.** Homologous removal of Neo Cassette (F3)	**Fwd:** GTA AGT CCC TGG AGA AGG GAG GGG GTT **Rev:** GAA CTC AGA GAC CCA CCC TCC	745bp (Targeted allele) 585bp (Wild type)
**Step 5.** PCR to identify pups after Exon 4 removal. Exon 4 is flanked by LoxP.	**Fwd:** CTG CAG AAG ACA CAA GCA GAG AGG A **Rev:** TGC TGT CCA CCA GTC ATG CTA	360bp (Targeted allele) 150bp (Wild type)
Generic Cre	**Fwd:** GCG GTC TGG CAG TAA AAA CTA TC **Rev:** GTG AAA CAG CAT TGC TGT CAC TT	100bp (Transgene)
Internal control	**Fwd:** CTA GGC CAC AGA ATT GAA AGA TCT **Rev:** GTA GGT GGA AAT TCT AGC ATC ATC C	324bp

### Confirmation of germline transmission (F1)

Male chimeric founders (F0) were crossbred with C57BL/6J females to confirm germline transmission. All of the F1 pups were genotyped by PCR. Although chimeric F1 pups were expected, the corrected agouti allele did not segregate with the floxed PKCε allele and pups carrying the targeted allele had either agouti or black fur. Few positive F1 were detected and most were produced after multiple litters had been sired. Two chimeric males produced positive F1 pups.

### Neo cassette removal (F2)

In order to remove the neomycin cassette and selection of PCKε-targeted clones, we crossbred male chimeric mice (F1) with female FLP recombinase mice (B6.Cg-Tg (ACTFLPe)9205Dym/J). Crossbreeding of these mice with chimeric F1 cause recombination between the Neo flanking FRT sites. All of the F2 pups were genotyped to confirm neo cassette removal and heterozygous floxed PKCε (PKCε^LoxP/+^) positive by PCR.

### Homozygous floxed PKCε (F3)

Eight week old heterozygous floxed PKCε (PKCε^LoxP/+^) male and female mice were crossbred to produce homozygous floxed PKCε (PKCε^LoxP/LoxP^) mice. Homozygous floxed PKCε pups were confirmed by genotyping using primers described in Table 1.

### The link of PKCε on prostate cancer growth

**Figure 2:**
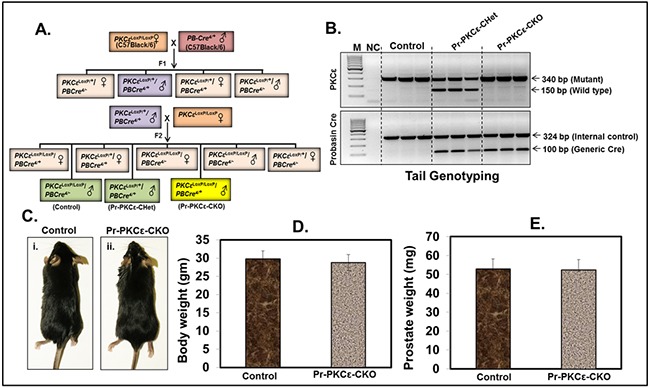
Characterization of floxed PKCε (PKCε^LoxP/LoxP^) mice using prostate specific Cre (PB^Cre+^) **A.** Breeding strategy to generate prostate specific conditional PKCε knockout mice. Female founders (F0) PKCε^LoxP/LoxP^ were crossbred with male prostate specific Cre transgenic (PB^Cre4/+^) mice. **B.** PCR gel picture showing genomic DNA tail genotyping for PKCε^LoxP/LoxP^, PKCε^LoxP/+^/PB^Cre4/+^ (Pr-PKCε-CHet), and PKCε^LoxP/LoxP^/PB^Cre4/+^ (Pr-PKCε-CKO) mice. **C.** Representative picture of nine week old control floxed PKCε and Pr-PKCε-CKO mice. Bar graph representing the body weight **D.** and prostate weight **E.** of control and Pr-PKCε-CKO mice. Value in bar graphs has shown the mean±SE of five mice in each group. No statistical significant difference was observed. Abbreviations: M=molecular weight marker; NC = negative control without DNA.

**Figure 3:**
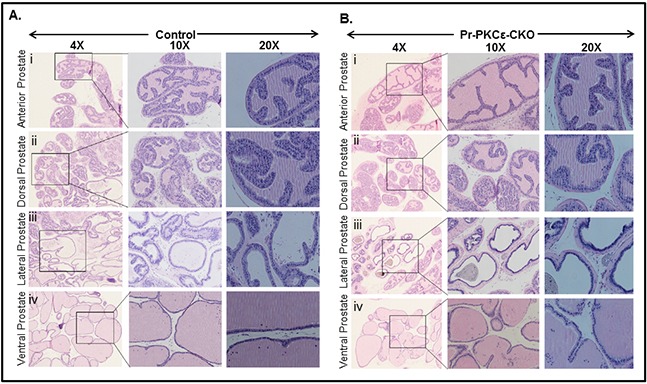
Histopathological analysis of PKCε^LoxP/LoxP^/PB^Cre4/−^ (Control) and PKCε^LoxP/LoxP^/PB^Cre4/+^ (Pr-PKCε-CKO) mice **A.** H&E staining of control mice anterior prostate (i), dorsal prostate (ii), lateral prostate (iii), and ventral prostate (iv). **B.** H&E staining of Pr-PKCε-CKO mice anterior prostate (i), dorsal prostate (ii), lateral prostate (iii), and ventral prostate (iv). The prostate of Pr-PKCε-CKO mice was indistinguishable from that of the control.

**Figure 4:**
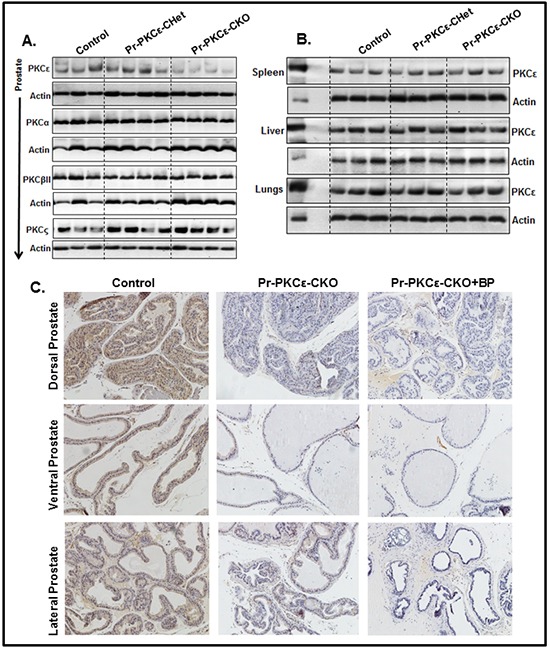
PKCε expression in the prostate of PKCε^LoxP/LoxP^/PB^Cre4/−^ (Control), and PKCε^LoxP/LoxP^/PB^Cre4/+^ (Pr-PKCε-CKO) mice **A.** Protein levels PKCε, PKCα, PKCβII and PKCζ in the prostate lysates of control PKCε-Het (PKCε^LoxP/+^/PB^Cre4/+^) and PKCε-KO PKCε^LoxP/LoxP^/PB^Cre4/+^ mice as analyzed by Western blot analysis. **B.** Protein level of PKCε in the spleen, liver and lungs (PKCε^LoxP/LoxP^), PKCε-Het (PKCε^LoxP/+^/PB^Cre4/+^) and Pr-PKCε-CKO PKCε^LoxP/LoxP^/PB^Cre4/+^ mice as analyzed by Western blot analysis. Fifty μg protein of each sample was loaded on the gel. Each lane of the blots in Figure 4A and Figure 4B represents an individual mouse sample. In Figure 4B, PC denotes the positive control where epidermal lysates (10 μg protein) from PKCε transgenic overexpressing mice (224) was loaded. Equal loading of protein was confirmed by stripping and re-probing the blots with an anti-β-actin antibody. **C.** Immunohistochemistry analysis of PKCε in the prostate tissues of PKCε^LoxP/LoxP^ (control), and PKCε-CKO PKCε^LoxP/LoxP^/PB^Cre4/+^ mice, and PKCε blocking peptide. Abbreviations: BP = blocking peptide.

To accomplish this, we generated novel prostate specific knockout (PKCε^LoxP/LoxP^/PB^Cre4/+^) (Pr-PKCε-CKO) mice using Cre-Lox recombination technology. Eight week old homozygous floxed PKCε (PKCε^LoxP/LoxP^) (control) crossbred with homozygous PB^Cre4/+^ mice to generate heterozygous and homozygous prostate specific Pr-PKCε-CKO mice. A brief out line of breeding scheme is shown in Figure 2A. Homozygous deletion of PKCε in pups of F2 generation was confirmed by genotyping (Figure 2B). Nine week old control (PKCε^LoxP/LoxP^) (n=8) and Pr-PKCε-CKO (n=8) mice from F2 generation were used for characterization. There were no phenotypic differences between the floxed PKCε and Pr-PKCε-CKO group's mice (Figure 2C–2D). Also no significant difference was observed in the prostate weight of control and Pr-PKCε-CKO group's mice (Figure 2A–2B). The prostate of both groups of mice showed no change as examined by histopathological analysis (Figure 3A–3B). Western blot analysis results showed reduced PKCε protein levels in the prostate of Pr-PKCε-CKO mice compared to control mice (Figure 4A). However, no change in the PKCε protein levels was observed in the spleen, liver and lungs of Pr-PKCε-CKO mice compared to wild type (Figure 4B) suggesting specific deletion of PKCε in the prostate. To determine whether deletion of PKCε in the prostate has any compensatory effects in Pr-PKCε-CKO mice, we analyzed other isoforms of PKC (PKCα, PKCβII, and PKCζ) in the prostate tissues of Pr-PKCε-CKO mice by Western blot analysis. No change was observed in the expression of other PKC isoforms in the prostate tissues of Pr-PKCε-CKO mice compared to wild type (Figure 4A) suggesting no compensatory effects on other isoforms of PKC. We further confirmed the inhibition of PKCε in the prostate tissues of Pr-PKCε-CKO mice by immunohistochemistry (Figure 4C). Results revealed inhibition of PKCε in the prostate epithelial cells of Pr-PKCε-CKO mice compared to wild type (Figure 4). PKCε immunostaining was confirmed by using blocking peptide of PKCε antibody (Figure 4C).

**Figure 5:**
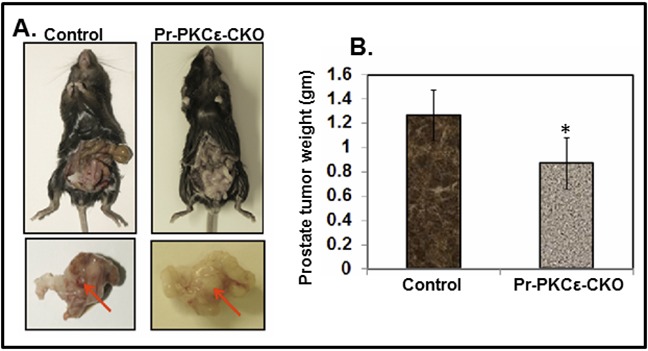
Inhibition of TRAMPC1 cells orthotopic xenograft tumors in prostate specific PKCε knockout (Pr-PKCε-CKO) mice **A.** Representative pictures of TRAMPC1 xenograft tumor bearing PKCε^LoxP/LoxP^ and Pr-PKCε-CKO mice. Representative pictures of excised urogenital apparatus of PKCε^LoxP/LoxP^ and Pr-PKCε-CKO mice at 8 weeks. Arrows indicate the development of xenograft tumors in the anterior prostate lobes. **B.** Bar graph showing prostate tumor weight of PKCε^LoxP/LoxP^ and Pr-PKCε-CKO mice. Values in bar graph represent mean±SE of 4 mice. P<0.05 was considered as significant value.

To determine the functional impact of prostate specific PKCε deletion, we performed an orthotopic xenograft study using TRAMPC1 cell line derived from transgenic adenocarcinoma of the mouse prostate (TRAMP) model [22]. The main objective of this experiment was to determine whether prostate specific deletion of PKCε influences the growth of TRAMPC1 cells derived xenograft tumors. In this experiment, a total of 8 mice Pr-PKCε-CKO (n=4) and floxed PKCε(n=4) were used and TRAMPC1 cells (2×10^6^) were implanted in the prostate. Both the group's mice were sacrificed at sixth week post-implantation. We observed a significant (P<0.05) decrease in the growth of prostate tumor weight compared to floxed PKCε mice (Figure 5A–5B).

### The link of PKCε to UVR-induced phosphorylation of Stat3

Chronic exposure to UVR is the major etiologic factor linked to the development of cSCC, a nonmelanoma form of skin cancer that can metastasize [23–27]. We have previously reported that PKCε levels in mouse epidermis dictates the susceptibility of mice to the development of cSCC elicited by UVR [28–35]. PKCε associates with Stat3 [15, 36]. Stat3 has two conserved amino acid residues (Tyr705 and Ser727) that are phosphorylated during Stat3 activation [15, 37]. PKCε phosphorylates Stat3 at Ser727. Stat3 is constitutively activated in both skin papilloma and carcinomas and is linked to cSCC [37].

**Figure 6:**
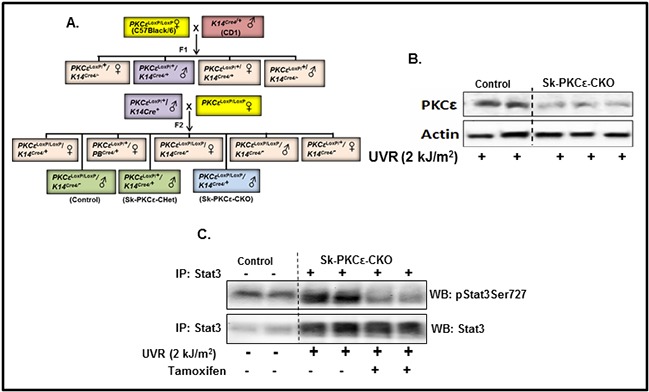
Specific deletion of PKCε in mouse epidermis inhibits phosphorylation of Stat3 in response to UVR irradiation **A.** Breeding strategy to generate skin specific conditional PKCε knockout mice. PKCε^LoxP/LoxP^ mice were crossbred with skin specific Cre transgenic (K14^Cre+^) mice to produce PKCε^LoxP/LoxP^/K14^Cre/+^ (Sk-PKCε-CKO) in F2 generation. PKCε^LoxP/LoxP^ (Control), PKCε^LoxP/+^/K14^Cre/+^ (Sk-PKCε-CHet), and PKCε^LoxP/LoxP^/K14^Cre/+^ (Sk-PKCε-CKO) mice were confirmed by genomic DNA tail genotyping as described in material and methods. Eight week old PKCε^LoxP/LoxP^ (n=4), and Sk-PKCε-CKO (n=4) mice were treated once with tamoxifen (75 mg/kg, i.p.) before UVR exposure (2 kJ/m^2^). Forty eight hours post-UVR treatment, mice were sacrificed and epidermal lysates prepared. **B.** PKCε expression in the epidermal lysates of tamoxifen treated, irradiated PKCε^LoxP/LoxP^ and Sk-PKCε-CKO mice as determined by Western blot analysis. Blots were stripped and reprobed for β-actin as a loading control. **C.** Expression of pStat3Ser727 and total Stat3 in the epidermal protein lysates of tamoxifen treated, irradiated, PKCε^LoxP/LoxP^ and Sk-PKCε-CKO mice as determined by immunoprecipitation (IP)/Western blot (WB) analysis. PC indicates the positive control where 10μg of epidermal protein lysates of PKCε transgenic overexpressing mice (224) was used.

To determine the link of PKCε to UVR-induced phosphorylation of Stat3, mice carrying a skin specific knockout of PKCε (PKCε^LoxP/LoxP^/K14^Cre/+^) (Sk-PKCε-CKO), were generated by cross breeding of eight week old floxed PKCε (PKCε^LoxP/LoxP^) with tamoxifen-inducible K14^Cre^ mice. A brief out line of breeding scheme is shown in Figure 6A. Homozygous deletion of PKCε in pups of F2 generation was confirmed by genotyping. Eight week old PKCε^LoxP/LoxP^ and PKCε^LoxP/LoxP^/K14^Cre/+^ (Sk-PKCε-CKO) mice were characterized. In this experiment, a total of nine mice (PKCε^LoxP/LoxP^) (n=3) and (Sk-PKCε-CKO) (n=6)) were used. Out of six Sk-PKCε-CKO mice three were administered a single dose of tamoxifen (75 mg/kg) i.p. All mice were were exposed once to UVR (2 kJ/m^2^). Forty eight hours post UVR treatment, mice were sacrificed and epidermal lysates were prepared. We first confirmed deletion of PKCε in the epidermis of Sk-PKCε-CKO mice by Western blot analysis. Results revealed reduced levels of PKCε protein in the epidermis of Sk-PKCε-CKO) mice compared to PKCε^LoxP/LoxP^ (Figure 6B). We determined the expression of pStat3Ser727 protein levels in the skin of tamoxifen untreated PKCε^LoxP/LoxP^, tamoxifen untreated Sk-PKCε-CKO, and tamoxifen-treated Sk-PKCε-CKO mice (Figure 6C). In this experiment we immunoprecipitated Stat3 in the protein lysates of these mice using Stat3 specific antibody and immunoblotted with pStat3Ser727 antibody. Western blot results demonstrated reduced Stat3 phosphorylation at Ser727 residue in tamoxifen-treated Sk-PKCε-CKO mice epidermis (Figure 6C). No change of Stat3 phosphorylation was observed in non-tamoxifen treated and untreated Sk-PKCε-CKO mice compared to PKCε^LoxP/LoxP^ mice (Figure 6C).

## DISCUSSION

PKCε, a novel PKC isoform is overexpressed in several human cancers and correlates with tumor aggressiveness [8, 15]. However, a genetic evidence linking PKCε to the induction, progression and metastasis of cancer in vivo is lacking. Furthermore, cancer growth and progression involve paracrine crosstalk between the tumor in the microenvironment and the cancer cells [38, 39]. A precise link of PKCε to the activation of stroma for tumor growth is also not known. This necessitated the generation of tissue-specific conditional PKCε knockout mice. We now present for the first time the generation and characterization of floxed PKCε mouse model using cre-lox technology (Figure 1). This mouse model will be essential tool to determine in vivo functional role and molecular mechanisms of PKCε linked to the induction and progression of various types of cancer.

Homozygous PKCε^LoxP/LoxP^ mice were generated on C57BL/6 background. Homozygous PKCε^LoxP/LoxP^ mice have normal body weight and phenotype. The effects of PKCε deletion in prostate and skin was determined by site specific deletion of PKCε using a prostate specific Cre (PB-Cre4+) and an epidermal specific Cre (K14 Cre) driver mice. The results of both Western blot and immunohistochemical analyses indicated tissue-specific deletion of PKCε. Cre-mediated tissue-specific deletion of PKCε affected neither body weight nor phenotype. No significant difference was observed in the prostate weight of PKCε^LoxP/LoxP^ and Pr-PKCε-CKO mice. Histopathological analyses of prostate from both PKCε^LoxP/LoxP^ and PKCε-Pr-CKO mice showed no pathology.

We have previously reported that constitutive deletion of PKCε in TRAMP mice inhibits spontaneous development of PCa [40]. These results imply that PKCε is linked to the induction of prostate cancer. However, in that model, PKCε was deleted in all tissues. In our study, we have shown that prostate specific deletion of PKCε inhibited the growth of TRAMP mouse tumor cells (TRAMPC1) in an orthotopic xenograft model. Thus, PKCε expression in the prostate epithelium is necessary for the growth of PCa cells derived xenografts tumors (Figure 5). These results indicate that knockdown of PKCε in the mouse prostate inhibits important growth factors and cytokines which are required for prostate tumor growth. We have previously reported that PKCε-mediated suppression of PCa in TRAMP mice accompanies inhibition of serum interleukin-6 (IL-6) levels [40]. The IL-6 is involved in tumor microenvironment. It may be the possibility that regression in orthotopic xenograft tumors in Pr-PKCε-CKO mice due to inhibition of IL-6.

To determine if PKCε is required for Stat3 phosphorylation at Ser727 in the epidermis, PKCε^LoxP/LoxP^ mice were bred to tamoxifen-inducible K14 Cre mice. PKCε deletion in the epidermis resulted in inhibition of ultraviolet radiation exposure (2 kJ/m^2^)-induced Stat3 phosphorylation, indicating that PKCε is required for this event in vivo in the skin.

In summary, PKCε is overexpressed in various cancers including non-small cell lung [41] and brain [42] cancers. Overexpression of PKCε transforms fibroblasts, colonic epithelial cells and LNCaP cells to androgen independence. PKCε level correlates with the aggressiveness of both breast and prostate cancer [8–15]. Future studies with the PKCε^LoxP/LoxP^ mice will be useful for defining the functional role and molecular mechanism of PKCε linked to various cancers in specific tissue, organ or cells.

## MATERIALS AND METHODS

### Chemicals and antibodies

Tamoxifen was purchased from Sigma Aldrich (Cat. # T5648). Monoclonal or polyclonal antibodies specific for actin, PKCε (sc214), PKCα (sc208), PKCζ (sc216), PKCβII (sc210) and Stat3 (sc20) were purchased from Santa Cruz Biotechnology (Santa Cruz, CA). Blocking peptides for PKCε antibody was also procured from Santa Cruz Biotechnology (sc214P). Monoclonal antibody for pStat3Ser727 (Cat. #612543) was purchased from BD Biosciences. Bacterial artificial chromosome (BAC) clones containing PKCε were purchased from Gene Service Ltd.

### Cell culture

Mouse prostate cancer cell line TRAMP-C1 (ATCC^R^ CRL-2730^TM^) was obtained from ATCC. These cell lines were extensively tested by ATCC for ampule passage number, population doubling time, post freeze viability, growth, morphology, mycoplasma contamination (agar and Hoechst DNA stain test), species determination (cytochrome C oxidase I gene assay, interspecies) and sterility test. These cells passed all above mentioned test used for the validity and authentication. We have propagated TRAMP-C1 cells from frozen stock that was authenticated by ATCC for above mentioned tests. Cells were used in the experiments just after two weeks in the lab. These cells were cultured in DMEM media containing 5% FBS, 5% Nu Serum, 10 nM dehydroisoandrosterone and 0.005 mg/ml bovine insulin.

### Mice

The targeting vector, PKCε mutant embryonic stem cells, and PKCε floxed mice were generated on the C57BL/6 background as described in Figure 1 at the University of Wisconsin Biotechnology Center's Transgenic Animal Facility. Homozygous floxed PKCε (PKCε^LoxP/LoxP^) mice were generated by intercrossing heterozygous floxed females and males. Removal of the neomycin cassette and selection of PCK-targeted clones, was achieved by crossbreeding male chimeric mice (F1) with female FLP recombinase mice (B6.Cg-Tg (ACTFLPe)9205Dym/J) that were obtained from Jackson Laboratory (Stock # 005703). All of the animal protocols were approved by the University of Wisconsin Research Animal Resources Committee in accordance with the NIH Guideline for the Care and Use of Laboratory Animals.

### Breeding strategies for generation of prostate specific conditional PKCε knockout mice

Transgenic PB^Cre4/+^ (Strain 01XF5, B6.Cg-Tg(Pbsn-cre)4Prb) mice were obtained from NCI Mouse Repository and maintained in our animal house facility at UW-Madison. To generate prostate specific knockout mice (PKC^LoxP/LoxP^/PB^Cre4/+^) (Pr-PKCε-CKO), homozygous floxed PKCε (PKCε^LoxP/LoxP^) female mice were crossbred with PB^Cre4/+^ male (C57BL/6) to produce PKCε^LoxP/+^/PB^Cre4/+^ (Pr-PKCε-CHet) (F1 generation). The male offspring of Pr-PKCε-CHet mice then crossbred with homozygous floxed PKCε (PKCε^LoxP/LoxP^) female to generate homozygous prostate specific conditional (PKC^LoxP/LoxP^/PB^Cre4/+^) Pr-PKCεCKO mice. PKCε floxed (PKC^LoxP/LoxP^)mice were screened for the floxed 340 bp and/or wild type 150 bp bands by PCR. Mice were genotyped for the Probasin-Cre (PB^Cre4/+^) transgene and an internal control by PCR. All of the primer sequences are given in Table 1.

### Histopathological examination

Prostate tissues of PKCε^LoxP/LoxP^ and PKCε^LoxP/LoxP^/PB^Cre4/+^ mice were excised and processed for histology as described previously [43]. Dr. Weixiong Zhong, a certified pathologist in the Department of Pathology, University of Wisconsin School of Medicine and Public Health, examined all of the tissue slides.

### Western blot analysis

We prepared whole tissue lysates of prostate, liver, lungs and spleen of PKCε^LoxP/LoxP^ and PKCε^LoxP/LoxP^/PB^Cre4/+^ mice as described previously [40]. Fifty micrograms of cell lysate were fractionated on 10-15% Criterion precast SDS-polyacrylamide gels (Bio-Rad Laboratories, Hercules CA). The fractionated proteins were transferred to 0.45 μm Hybond-P polyvinylidene difluoride (PVDF) transfer membrane (Amersham Life Sciences, Piscataway NJ). The membrane was then incubated with the specific antibody followed by the appropriate horseradish peroxidase-conjugated secondary antibody (Thermo Scientific, Pittsburgh, PA). The detection signal was developed with Amersham's enhanced chemiluminescence reagent and using FOTO/Analyst Luminary Work Station (Fotodyne Inc., Hartland, WI). The Western blots were quantified by densitometric analysis using Total lab Nonlinear Dynamic Image analysis software (Nonlinear USA, Inc., Durham, NC).

### Immunohistochemistry

The paraffin embedded sections (4mm thickness) of excised prostate tissues of PKCε^LoxP/LoxP^ and PKCε^LoxP/LoxP^/PB^Cre4+^ mice were deparaffinized by placing the slides at 60°C for 2 hours followed by 3 changes of Xylene for 10 minutes each. Slides were placed in 0.3% methanol/Hydrogen peroxide for 20 minutes for quenching endogenous peroxidase. Slides were rehydrated in one change of absolute, 95%, 75%, and 50% ethanol and distilled water. Antigen retrieval was performed by incubating samples at 116°C for 15 seconds in the decloaking chamber by using a Tris-urea solution (pH 9.5). After antigen retrieval, tissues slides were incubated with 2.5% normal horse serum (R.T.U. Vectastain Universal Elite ABC Kit, Vector Laboratories, Burlingame, CA) for 20 minutes to block non-specific binding of the antibodies. Subsequently, the slides were incubated overnight with a mixture of PKCε (1:500) dilution in normal antibody diluents (Scy Tek # ABB-125, Logan, UT) in a humidified chamber. Specificity of immunostaining of these proteins was confirmed by using blocking peptide of PKCε (served as a negative control). The mixture of antibodies was decanted and the slides were washed three times in TBS (pH7.4). The slides were incubated with appropriate secondary antibodies for 30 minutes at room temperature. Slides were rinsed with TBS for 5 min and ABC reagent (Vector kit) was applied for 30 minutes. Immunoreactive complexes were detected using DAB substrate (Thermo Scientific, Pittsburgh, PA), and counter stained by using hematoxylin (Fischer Scientific, Pittsburgh, PA) for nuclear staining. Finally, slides were mounted with a cover slip using mounting medium. All sections were visualized under a Zeiss-Axiophot DMHT microscope and images captured with an attached camera.

### Breeding strategies for generation of epidermal specific conditional PKCε knockout mice

Transgenic tamoxifen-inducible keratin 14^Cre/+^ mice (Tg (KRT14-cre/ERT)20Efu/J) mice were purchased from Jackson laboratory. To generate epidermal specific PKCε knockout (PKCε^LoxP/LoxP^/K14^Cre/+^) mice, we crossbred heterozygous PKCε^LoxP/LoxP^ female mice with homozygous male keratin 14Cre (K14^Cre^) mice to produce PKCε^LoxP/+^/K14^Cre/+^ (PKCε-Het) (F1 generation). The offspring of PKCε-Het mice then crossbred with homozygous floxed PKCε (PKCε^LoxP/LoxP^) female to generate homozygous (PKCε^LoxP/LoxP^/K14^Cre/+^) mice. These offspring (F2) were genotyped by RT-PCR for floxed PKCε and generic cre using primers listed in Table 1.

### Characterization of epidermal specific conditional PKCε knock out mice

A total of nine (8 week old) PKCε^LoxP/LoxP^/K14^Cre/+^ mice were used for characterization. Three mice (PKCε^LoxP/LoxP^/K14^Cre/+^) were administered with a single dose of tamoxifen (75 mg/kg) i.p. followed by single dose of UVR exposure (2 kJ/m^2^). Forty eight hours post-treatment, mice were sacrificed and epidermal lysates were prepared for immunoprecipitation/Western blot analysis as described [15, 44].

### Orthotopic xenograft

Ten weeks old homozygous floxed PKCε (PKCε^LoxP/LoxP^) (n=4) and prostate specific conditional knockout (PKCε^LoxP/LoxP^/PB^Cre4/+^) (n=4) were used for the xenograft study. To establish orthotopic xenografts in these mice, TRAMPC1 cells (2.0 × 10^6^) were suspended in 20 μl of HBSS media and directly implanted into the prostate. Six week post-implantation of TRAMPC1 cells, all of the mice were sacrificed and examined for prostate tumor growth. Weight of each mouse excised prostate tumor was recorded [45].

### Statistical analysis

Student's t test was carried out to determine the significance. The p value < 0.05 was considered as significant.

## Abbreviations

PC: Prostate cancer
PKCε: Protein kinase C epsilon
PKCε-CKO: conditional PKCε knockout mice
TRAMPC1: Transgenic adenocarcinoma of the mouse prostate cells
